# Radiolytic H_2_ Production in Martian Environments

**DOI:** 10.1089/ast.2017.1654

**Published:** 2018-09-12

**Authors:** Mary Dzaugis, Arthur J. Spivack, Steven D'Hondt

**Affiliations:** Graduate School of Oceanography, University of Rhode Island, Narragansett, Rhode Island.

**Keywords:** Mars, Water radiolysis, Habitability, Hydrogen, Geochemistry, Mars 2020

## Abstract

Hydrogen, produced by water radiolysis, has been suggested to support microbial communities on Mars. We quantitatively assess the potential magnitude of radiolytic H_2_ production in wet martian environments (the ancient surface and the present subsurface) based on the radionuclide compositions of (1) eight proposed Mars 2020 landing sites, and (2) three sites that individually yield the highest or lowest calculated radiolytic H_2_ production rates on Mars. For the proposed landing sites, calculated H_2_ production rates vary by a factor of ∼1.6, while the three comparison sites differ by a factor of ∼6. Rates in wet martian sediment and microfractured rock are comparable with rates in terrestrial environments that harbor low concentrations of microbial life (*e.g.*, subseafloor basalt). Calculated H_2_ production rates for low-porosity (<35%), fine-grained martian sediment (0.12–1.2 n*M*/year) are mostly higher than rates for South Pacific subseafloor basalt (∼0.02–0.6 n*M*/year). Production rates in martian high-porosity sediment (>35%) and microfractured (1 μm) hard rock (0.03 to <0.71 n*M*/year) are generally similar to rates in South Pacific basalt, while yields for larger martian fractures (1 and 10 cm) are one to two orders of magnitude lower (<0.01 n*M*/year). If minerals or brine that amplify radiolytic H_2_ production rates are present, H_2_ yields exceed the calculated rates.

## 1. Introduction

The search for extraterrestrial life has prompted >20 surface and orbital missions to Mars (Walter, 2015). Many of these missions have focused on identifying past and present aqueous environments, as water is necessary for life as we know it. However, it is not the only requirement for life. Habitable environments must also provide biologically harvestable energy. A variety of geochemical processes produce chemical species that can be metabolized by microorganisms for energy. Several studies have proposed that some subsurface microbial communities on Earth rely on molecular hydrogen (H_2_) as their primary electron donor (*i.e.*, Stevens and McKinley, 1995; Pedersen, 2000). In terrestrial subsurface environments, H_2_ is abiologically produced by water radiolysis (*i.e.*, Pedersen, 2000; Lin *et al.*, 2005a; Blair *et al.*, 2007; Türke *et al.*, 2015; Dzaugis *et al.*, 2016), rock weathering (*i.e.*, Stevens and McKinley, 1995; Pedersen, 2000), and serpentinization (*i.e.*, Kelley *et al.*, 2001, 2005). These chemolithotrophic communities serve as models for life on other planets, such as Mars (McCollom and Seewald, 2013). Here we quantify the potential for radiolytic production of H_2_ in water-saturated environments, such as the ancient martian surface or the present subsurface.

Previous studies suggested that radiolysis might support life on Mars (*i.e.*, Lin *et al.*, 2005b; Sherwood Lollar *et al.*, 2007; Lefticariu *et al.*, 2010; Türke *et al.*, 2015). However, few studies quantified radiolysis rates. To better understand the origin of the subsurface methane flux on Mars, Onstott *et al.* (2006) investigated the diffusion of He, H_2_, and CH_4_ through the martian crust. They calculated radiolytic production rates for the deep subsurface crust based on the chemical composition of martian meteorites and a 6.6 km-thick crustal profile of estimated porosity. In contrast to their global approach, we calculate specific rates for a broad range of lithologies, including both water-saturated rock and sediment, based on compositional data at 11 specific sites on Mars.

These 11 sites include the three currently proposed Mars 2020 Rover landing sites (Witze, 2017), five other sites that were previously under consideration for the Mars 2020 mission (Ono *et al.*, 2016), and three sites with minimum or maximum mean martian rates of radiolytic H_2_ production (based on radionuclide concentrations). Our purpose of selecting these sites is to quantify (1) the magnitude of radiolytic H_2_ production in the wet martian past at potential Mars 2020 landing sites, and (2) the range of H_2_ production rates in present-day martian subsurface groundwater.

Identification of past and present habitable environments is one of the main objectives of Mars Exploration missions (Mustard *et al.*, 2013). Because oxidative H_2_ respiration is a source of chemical energy for microbial life, estimates of past H_2_ production rates based on the radioisotope compositions of proposed Mars 2020 sites are important for understanding the ancient habitability of the sites.

Eight sites were originally considered as the possible landing sites for the Mars 2020 mission (Mustard *et al.*, 2013). Many of these locations have similar geological and topographic features, in part because the final site must be safe for rovers to land and travel (Mustard *et al.*, 2013). The eight candidate sites were narrowed to three based on the following criteria: an ancient environment likely to have been habitable, accessible rocks with potential for biosignature preservation, and geological materials that may answer questions about planetary evolution (Witze, 2017).

The three sites currently under consideration for the Mars 2020 landing site are as follows: Jezero Crater, an ancient lake that has both deltaic and lacustrine deposits with well-defined stratigraphy (Schon *et al.*, 2012; Witze, 2017); NE Syrtis Major which was once volcanically active, with widespread evidence of past liquid-water activity and a variety of interesting environments, including a banded olivine-carbonate unit and a crater-retaining cap of mafic rock (Hiesinger and Head, 2004; Ono *et al.*, 2016); and Columbia Hills, where Mars Exploration rover Spirit provided evidence of ancient hydrothermal activity and/or hot springs (Squyres *et al.*, 2008).

In addition to these three locations, we evaluate radiolytic H_2_ production for five other sites originally under consideration for Mars 2020: Eberswalde Crater, Holden Crater, Nili Fossae, Mawrth Vallis, and SW Melas Chasma. We include these sites as points of comparison for the three sites still under consideration, as all eight sites may have once harbored liquid water, and potentially H_2_-oxidizing life (Ono *et al.*, 2016).

**FIG. 1.**
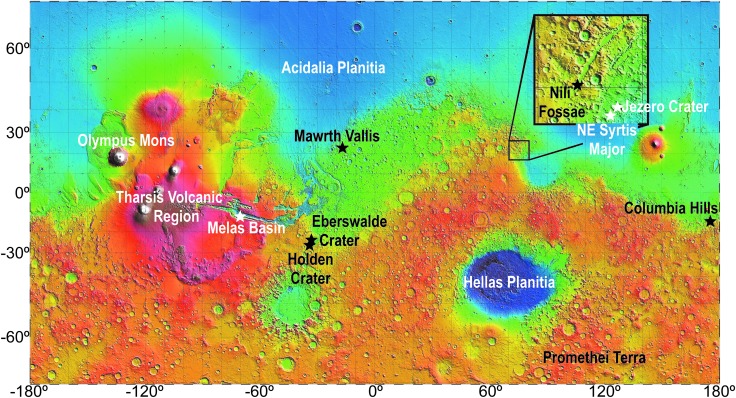
Topographic map of Mars. Site names and significant features identified. Topology map based on data from the Mars Orbiter Laser Altimeter (Smith *et al.,* 2003).

Finally, we chose three additional locations, based on their radioisotope compositions, to evaluate minimum and maximum radiolytic H_2_ production rates on the past martian surface and in the present subsurface: Acidalia Planitia (maximum rate), the northern pole (minimum rate for an ice-covered region), and Promethei Terra (minimum rate for a region without ice cover). The locations of the 11 sites are shown in Figure 1.

Martian surface material ranges from loose, fine-grained regolith to large pieces of fractured rock. To account for this heterogeneity, we calculate H_2_ production rates using two different radiolysis models. One model is for fractured hard-rock aquifers (Dzaugis *et al.*, 2015, 2016), the other is for water-saturated fine-grained sediment (Blair *et al.*, 2007).

## 2. Methods

In this section, we describe the radiolysis models and discuss the variables used in our calculations: fractured rock composition, sediment porosity, radioactivity, density, and H_2_ yield per 100 eV absorbed (*G*-value). Lithologies of the 11 localities are described in Table 1, and calculated H_2_ production rates are given in Table 2.

**Table 1.** Locations, Radioisotope Concentrations, and Lithologic Descriptions of Sites Used to Calculate Potential Martian Radiolytic H_2_ Production Rates

*Site*	*Location (lat °N, long °E)*	*Thorium (ppm)*	*Potassium (wt %)*	*Main lithology/geological feature*
Acidalia Planitia	48, −32	0.94	0.44	Mottled lava plains
Mawrth Vallis	24.0, −18.9	0.74	0.35	Channel deposits, phyllosilicate minerals
**Columbia Hills**	−**14.4, 175.6**	**0.63**	**0.32**	**Hydrothermal, hot springs**
**NE Syrtis Major**	**17.8, 77.1**	**0.60**	**0.31**	**Volcanic and hot spring activity**
**Jezero Crater**	**18.5, 77.4**	**0.59**	**0.31**	**Ancient delta rich in clay minerals**
Nili Fossae	21.0, 74.5	0.57	0.29	Methane plumes, carbonate, and altered clay minerals
Holden Crater	−26.4, −34.9	0.53	0.28	Ancient lake and flood deposits, alluvial fans
Eberswalde Crater	−23.0, −33.0	0.51	0.30	Ancient delta, possible lake deposits
SW Melas Basin	−12.2, −70.0	0.45	0.31	Canyon, cuts through lake deposits
Promethei Terra	−67, 97	0.25	0.22	Interbedded volcanic rock and impact breccia
Northern pole	87, 5	0.17	7.7E-02	Dust and ice

Radionuclide concentrations are determined from the interpolated distribution maps in Figure 1. Uranium concentrations are calculated using a U/Th ratio of 0.28. **Bold** font marks the current sites under consideration for Mars 2020 rover landing.

**Table 2.** Radiolytic H_2_ Production Rates Given the Radionuclide Concentrations of 11 Localities on Mars' Surface

*Site*	*Main lithology/geological feature*	*H_2_ production rates for water-filled rock fractures in n*M*H_2_/year*			*H_2_ production rates for water-saturated sediment in n*M*H_2_/year*		
		*Width: 1 μm*	*Width: 1 cm*	*Width: 10 cm*	*Porosity: 5%*	*Porosity: 35%*	*Porosity: 80%*
Acidalia Planitia	Mottled lava plains	0.35	0.008	0.003	1.2	0.71	0.19
Mawrth Vallis	Channel deposits, phyllosilicate minerals	0.27	0.007	0.003	0.93	0.56	0.15
**Columbia Hills**	**Hydrothermal, hot springs**	**0.23**	**0.006**	**0.002**	**0.80**	**0.48**	**0.13**
**NE Syrtis Major**	**Ancient delta rich in clay minerals**	**0.22**	**0.006**	**0.002**	**0.75**	**0.45**	**0.12**
**Jezero Crater**	**Volcanic and hot spring activity**	**0.22**	**0.006**	**0.002**	**0.75**	**0.45**	**0.12**
Nili Fossae	Methane plumes, carbonate, and altered clay minerals	0.21	0.005	0.002	0.72	0.43	0.11
Holden Crater	Ancient lake and flood deposits, alluvial fans	0.20	0.005	0.002	0.67	0.40	0.10
Eberswalde Crater	Ancient delta, possible lake deposits	0.19	0.005	0.002	0.65	0.39	0.10
SW Melas Basin	Canyon, cuts through lake deposits	0.17	0.005	0.002	0.58	0.35	0.09
Promethei Terra	Interbedded volcanic rock and impact breccias	0.10	0.003	0.001	0.33	0.20	0.05
Northern pole	Dust and ice	0.06	0.002	<0.001	0.21	0.12	0.03

Volume-normalized rates are given for three fractured-rock scenarios (varying fracture width) and three sediment scenarios (varying porosity). **Bold** font marks the current sites under consideration for Mars 2020 rover landing.

### 2.1. Fractured hard-rock model

We previously used this model to calculate production rates in terrestrial oceanic basaltic aquifers (Dzaugis *et al.*, 2016). In this study, we use parameter values appropriate for Mars.

Within hard-rock aquifers, ionizing radiation produced during the decay of radioactive elements in the rock decomposes water molecules and leads to production of hydrogen molecules (Le Caër, 2011). To calculate H_2_ production rates within fractured hard-rock aquifers, we first integrate the decay energy that reaches the water [MeV/(cm^2^·s)]. This is the radiant flux, and depends on

- Decay power, decay energy per time per solid angle [MeV/(sr·s)], determined by the activity decay energy of the radionuclides (U, Th, and K) in the rock.
- Irradiance, amount of incident power normalized to surface area (decay power/cm^2^), depends on the geometry of the radiation's path from the source to the surface of interest.
- Attenuation, the decrease of intensity as the radiation travels through rock and water.

The water-volume-normalized dose rate [MeV/(cm^3^_water_·s)], is equal to the radiant flux divergence between rock–water interfaces divided by the distance between the interfaces. Finally, H_2_ production rate (nanomolar H_2_/year) is given as follows:

\begin{align*}
{ \rm{Production}} \;{ \rm{rate }} = ( {G_ \alpha }{D_ \alpha } + {G_ \beta }{D_ \beta }+{G_ \gamma }{D_ \gamma } ) ,
\end{align*}

where the absorbed dose rate (*D*_α,β,γ_) from each type of radiation is multiplied by its respective *G*-value (*G*_α,β,γ_), and the number of H_2_ molecules created per 100 eV of energy absorbed (see Section 2.5 for more details on *G*-values). For a more detailed description of the parameters and the equations, see Dzaugis *et al.* (2015).

As mentioned above, the radiant flux and therefore dose rates are affected by the attenuation of radiation energy. Attenuation depends on matrix composition. The martian crust is dominantly basaltic (McSween, 2015). Igneous rocks from Gusev and Gale craters and SNC (Shergotty, Nakhla, and Chassigny) meteorites, as well as measurements from the γ ray spectrometer (GRS) on Mars Odyssey, all indicate ∼40–55 wt % SiO_2_ (McSween *et al.*, 2003). We thus utilize energy–range relationships for each type of radiation traveling through basalt or water. For α-radiation, the energy–range equation for basalt was derived by Brennan and Lyons (1989). To calculate attenuation of β and γ radiation through rock and water, we use energy–range data from the National Institute of Standards and Technology (NIST) database (Hubbell and Seltzer, 2004; Berger *et al.*, 2005). Because the NIST database does not include energy–range data for basalt, we use data for SiO_2_ adjusted for the higher density typical of martian basalt.

The dose rate also depends on the thickness of rock adjacent to the fracture. We calculate dose rates produced from the radiation emitted by 1-m-thick rock abutting both sides of the fracture. One meter of rock accounts for >99% of the entire dose rate. α- and β-Radiation generated by the ^238^U, ^235^U, and ^232^Th decay series and β particles by ^40^K decay have ranges which are much <1 m in basalt, and >99% of γ-radiation is absorbed over this distance. However, since α-radiation is more energetic than β- or γ-radiation and travels in rock on average ∼30 μm, absorbed dose rates in microfractures produced by 30 μm-thick rock are only ∼10% lower than rates from 1 m of rock.

Using the dose rates and *G*-values, we calculate water-volume-normalized radiolytic H_2_ production rates for a range of fracture apertures. H_2_ production rate per volume of water decreases as fracture width increases. The stopping power of water results in loss of particle energy near the rock–water interface. The decrease in volume-normalized H_2_ production is due to the limited ranges of α and β radiation (30 μm and a few mm, respectively) and increased volume of water (Dzaugis *et al.*, 2016), which does not produce significant radiation itself. Since we do not know the distribution of fracture widths in martian basalt, we present three cases to illustrate how H_2_ yields depend on width (1 μm, 1 cm, and 10 cm). Supplementary Table S1 (Supplementary Data are available online at www.liebertonline.com/ast) summarizes factors relevant to H_2_ calculations.

### 2.2. Sediment model

We also calculate radiolysis rates for fine-grained water-saturated sediment in which we assume a homogeneous mixture of water and particles <30 μm in diameter (stopping distance of α-particles, the distance a particle travels before its kinetic energy is 0) (Blair *et al.*, 2007).

In this protocol, dose rates for α-, β-, and γ-radiation are calculated based on activity (Section 2.3), the sum of radiative energy released by each radionuclide decay series, grain density (Section 2.4), the ratios of relative stopping power for each radiation type, and porosity of the sediment (Blair *et al.*, 2007). Once radiation doses are known, we can calculate the H_2_ production by multiplying the dose rate from α-, β-, and γ-radiation by their respective radiation chemical yields (*G*-value, see Section 2.5). For a full summary of the factors used in this protocol, see Supplementary Tables S2 and S3.

We include three different porosities (5%, 35%, and 80%) to parallel the fractured rock model. The calculations with 5% porosity define a high-H_2_-production end-member scenario, because H_2_ production rates per volume of water are highest where porosity is low. The calculations with 35% porosity are consistent with many previous studies of martian sediment. A value of 35% porosity has been used in many studies that calculate timescales for formation of martian depositional fans and deltas, such as in channelized fans located in Holden crater and Melas Chasma (Jerolmack *et al.*, 2004; Kleinhans, 2005; Metz *et al.*, 2009). Finally, because our model is restricted to medium-silt grain size (30 μm), we provide results for 80% porosity, a value typical of uncompacted pelagic clay on Earth's seafloor (Hamilton, 1976; Spinelli *et al.*, 2004).

We update the values of Blair *et al.* (2007) for the decay energy of the ^238^U, ^235^U, and ^232^Th series, assuming secular equilibrium, and ^40^K (Supplementary Table S4). For each radionuclide, we separately sum the energy of α-, β-, and/or γ-radiation energy (U and Th: MeV/decay series; K: MeV/decay). Our β-energy sums differ greatly from those of Blair *et al.* (2007), because we calculate an average initial energy for each β-particle, while Blair *et al.* use the maximum energy value for each β-particle. β-Particles are emitted with a continuous spectrum of energies ranging from near-zero to a maximum value specific to each radionuclide. To account for this distribution, when calculating β-decay energy sums, we use the average initial energy, which is approximately one-third of the maximum energy (L'Annunziata, 2007). These updated values match the values that we use in our fractured rock calculations.

### 2.3. Radionuclide concentrations

Both radiolysis models require radionuclide activities. The principle radioisotopes that can drive water radiolysis on Mars are in the ^238^U, ^235^U, and ^232^Th decay series and ^40^K. The U and Th series emit α-, β-, and γ-radiation, whereas ^40^K emits β- and γ-radiation. Radiolysis rate is proportional to radionuclide activities. For ^40^K and ^232^Th abundances, we used data collected using the GRS on the Mars Odyssey (Boynton *et al.*, 2007). GRSs determine the abundance and distribution of K and Th by measuring characteristic γ rays that are emitted from the top few tens of centimeters of the planet's surface (Boynton *et al.*, 2004). The GRS data comprise the most exhaustive record of radionuclide concentrations for Mars. However, this record is geographically low resolution. The footprint diameters over which 50% of the Th and K signals are received are 240 and 215 km, respectively. Therefore, although microscale variability in radionuclide concentrations undoubtedly exists, our calculations do not take into account variations in radionuclides at distance smaller than the GRS footprints.

Landers and rovers have also collected K abundance data (McLennan, 2001). The lander and rover measurements, however, are more spatially restricted, and U and Th concentrations are not determined. K abundance based on satellite GRS correlates to *in situ* data collected at landing sites by surface instruments (Boynton *et al.*, 2007; Karunatillake *et al.*, 2007).

Galactic cosmic rays (GCRs) can also contribute to radiolysis at the surface of Mars, if water is present. GCRs comprise 85% protons, 14% α particles, and small percentage of heavy ions (Dartnell *et al.*, 2007). However, ∼3 m below the surface GCR are no longer the main source of radiation; at depths >3 m, radionuclides contained in martian sediment or rock provide the main radiation source for radiolytic H_2_ production (Hassler *et al.*, 2014).

**FIG. 2.**
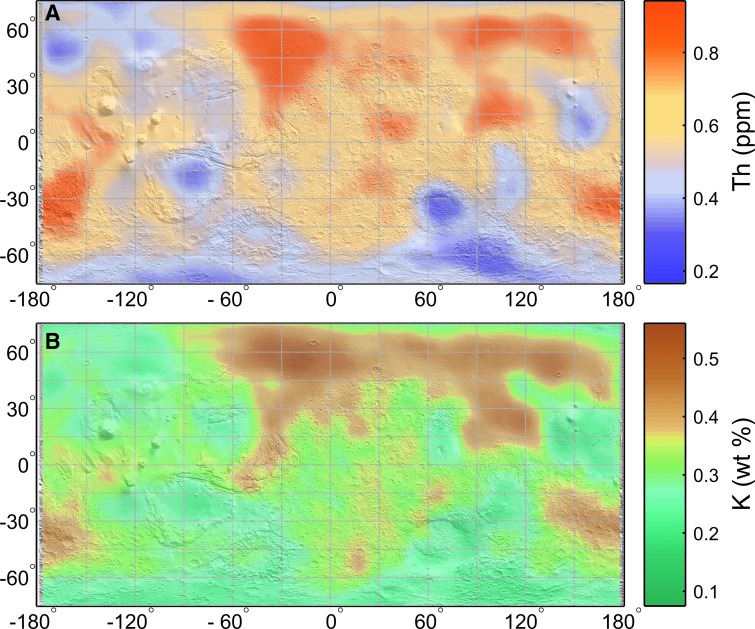
Radionuclide concentrations. **(A)** Thorium (ppm) and **(B)** Potassium (wt %). Th and K concentrations are based on results from the Mars Odyssey Gamma Ray Spectrometer (Boynton *et al.*, 2007). As described in the text, we calculate U concentrations using a constant U/Th ratio of 0.28.

Th and K are mapped for the globe (Boynton *et al.*, 2007). However, near the poles, ice cover can bias Th and K abundances toward lower values because some of the top 10 cm is composed of ice (Taylor *et al.*, 2007; Boynton *et al.*, 2007). Boynton *et al.* (2007) summed elemental spectra and binned them in 5° × 5° bins to improve signal-to-noise ratios. We include the reported uncertainty associated with smoothed radionuclide concentration measurements in our final radiolysis error calculations reported in Supplementary Tables S5 and S6. U abundances have not been calculated from GRS data due to its weak signal-to-noise ratio (Boynton *et al.*, 2007; Karunatillake *et al.*, 2007). In this study, we calculate U concentrations by using Th concentrations from GRS data and assuming a constant U/Th ratio of 0.28 (a value characteristic of most SNC meteorites) (McLennan, 2003; Baratoux *et al.*, 2011). We show global maps of U, Th, and K distributions in Figure 2.

Radionuclide concentrations vary with lithology. There is evidence on Mars for a global dust unit with relatively constant basaltic mineralogy (Yen *et al.*, 2005). However, if this global unit is present, it is <10 cm thick since the martian surface does not have a constant radionuclide distribution as measured by GRS (Boynton *et al.*, 2007).

### 2.4. Density

Density values are based on the range suggested by Baratoux *et al.* (2014) for martian crust. Baratoux *et al.* (2014) based their calculations of density on GRS surface element measurements, major element chemistry of martian meteorites, and chemical analysis of martian igneous rocks by rovers. They argue that the basaltic component of the martian crust has a density >3100 kg/m^3^, which is higher than previously thought (Baratoux *et al.*, 2014; Zharkov and Gudkova, 2016). In our calculations, we use density of 3350 kg/m^3^ which is the peak value calculated using the CIPW (Cross, Iddings, Pirsson, and Washington) norm on GRS chemical maps (Baratoux *et al.*, 2014). While this density is representative of martian basalt, it is also similar to values used in sediment transport models for Mars which use grain density values of 3400 km/m^3^ (Kleinhans, 2005; Hoke *et al.*, 2014).

### 2.5. *G*-values

We calculate H_2_ production rates using pure water *G*-values (Table 3). These *G*-values are well constrained for each type of radiation. For both our models, we assume constant *G*-values for the energy ranges of radiation in this study (<5.8 MeV). *G*-values depend on linear energy transfer (LET), the energy absorbed by the medium normalized to distance (−dE/dx). However, *G*(H_2_) appears to plateau at a LET value of ∼150 keV/μm, which corresponds to a 5 MeV α-particle (Crumière *et al.*, 2013). Therefore, at high LET values of α-particles in this study, there is minimal effect on the *G*-value. We did not find any work specifically on the effect of β-particle LET, therefore we assume yields to be constant for *G*_β_(H_2_) as well. The *G*(H_2_) values we use in our calculations provide a minimum estimate of H_2_ production because specific minerals (*e.g.*, Kumagai *et al.*, 2013) and/or brines (Kelm and Bohnert, 2004; Kumagai *et al.*, 2013) can increase H_2_ yields. In Section 3.4, we further discuss potential effects of minerals and brine on *G*-values and H_2_ production rates. As mentioned previously, our calculations normalize the final radiolytic H_2_ production values to the total volume of water, presented in units of nanomolar H_2_ per year (n*M*/year).

**Table 3.** *G*-Values in Molecules H_2_/100 eV for Pure Water

	*α*	*β*	*γ*
*G*(H_2_)	1.30^a^	0.6^b^	0.24^a^

^a^ Essehli *et al.* (2011).

^b^ Kohan *et al.* (2013).

## 3. Results

As we mentioned previously, fracture width and porosity affect water-volume-normalized H_2_ production rate (nM/year), principally because radioactive element concentrations in water are typically much lower than those in rock. We assume that concentrations of U, Th, and K in martian water are equivalent to those in seawater. These concentrations are so low that we do not include them in our calculations (Pilson, 2012). Volcanic rocks have a range of fracture widths. In fractured rock, with a constant radioisotope composition, microfractures have the highest H_2_ production rates per volume of water (Table 2) (Dzaugis *et al.*, 2016). Production rates in a 1 μm-wide fracture are more than two orders of magnitude higher than those in a 10 cm-wide fracture (Table 2). H_2_ production rates (n*M*/year) in sediment at 5% porosity are ∼6.4 times higher than those at 80% porosity (Table 2). Production rates in low-porosity, fine-grained sediments are higher than those in fractured rock. At any given site, H_2_ production rate in sediment with ∼60% porosity is similar to the rate in microfractured rock.

H_2_ production rates for water-saturated lithologies with radionuclide concentrations equal to our 11 sites are summarized in Table 2. Calculated H_2_ production rates are within a factor of 2 of each other, given constant physical conditions, and the GRS radioisotope compositions of the original eight potential Mars 2020 landing sites. We also calculated uncertainties for H_2_ production, based on reported one-sigma uncertainty, the estimated standard error associated with the smoothed GRS radionuclide data (Boynton *et al.*, 2007). The relative uncertainty for most sites ranges from 5% to 8%, although Promethei Terra and the Northern Pole have uncertainties closer to 15% (Supplementary Tables S5 and S6).

Mawrth Vallis, an ancient channel, has the highest rates of the landing sites, while SW Melas basin rates are ∼1.6 times lower (Table 2). The production rates for the three sites still under consideration for the Mars 2020 mission (Jezero Crater, NE Syrtis Major, and Columbia Hills), which have the next highest rates, are within 7% of each other. Across the 11 sites in this study, calculated production rates vary by a factor of ∼6. This range includes the likely low-biased radionuclide concentrations at latitudes >75°N due to the presence of ice. If ice-covered regions are excluded, the range is ∼3.5-fold.

Acidalia Planitia, in the northern highlands, has the highest production rates due to its high radioisotope abundances (Table 2). Fine-grained sediments with 5% porosity have rates of 1.2 n*M*/year, whereas microfractured rock has a H_2_ production rate of 0.35 n*M*/year, assuming the same composition at a depth where water is now present or was present in the past. Between 75°N and 75°S, the radioisotope composition of Promethei Terra has the lowest calculated rates: 0.33 n*M*/year for 5% porosity sediment and 0.10 n*M*/year for 1 μm-wide fractures. Including polar regions, H_2_ production rates may be as low as 0.21 n*M*/year in 5% porosity sediment and 0.061 n*M*/year in 1 μm-wide fractures (Table 2).

## 4. Discussion

In the following sections, we discuss martian H_2_ production rates and assess how much life might be supported by radiolysis. We then compare martian and terrestrial rates. Because we assume water saturation, the calculated rates do not hold for present surface conditions at these sites. Instead, they correspond to rates for ancient (wet-surface) Mars and present-day wet subsurface environments with the same radioisotope compositions and physical properties used in our calculations.

Previous studies identified the potential for chemolithotrophic life in the martian subsurface due to the likely presence of liquid water and the production of reduced chemicals (primarily H_2_ through serpentinization of ultramafic rock) (Mancinelli, 2000; Ehlmann *et al.*, 2010; McCollom and Seewald, 2013). Most production of H_2_ by serpentinization is limited to temperatures of 150°C to 315°C (McCollom and Bach, 2009). In contrast, radiolytic H_2_ production is more ubiquitous, occurring wherever water or ice is in contact with rock or sediment.

### 4.1. H_2_ production rates at eight proposed landing sites

Calculated production rates for water-saturated lithologies are all within a factor of 2 for the eight landing sites. The relative uncertainties in production rates are similar, ∼7%, at these sites (Supplementary Tables S5 and S6). These uncertainty estimates include the reported uncertainty of GRS radionuclide data. Of the three sites currently under consideration for the Mars 2020 mission, radionuclide concentrations characteristic of Columbia Hills lead to H_2_ production rates that are slightly higher than those at the other two sites. However, considering the uncertainty of the GRS measurements at these three sites, the three values are indistinguishable from each other (Supplementary Tables S5 and S6). Of the other five sites previously under consideration for Mars 2020, the radioisotope concentrations of Mawrth Vallis yield the highest production rates and the concentrations of SW Melas yield the lowest rates (Table 2). For fine-grained sediment with 5% porosity, Mawrth Vallis production approaches 1 n*M*/year. The highest rates based on the fractured rock model are three to four times lower.

If radiolytic H_2_ supported life on the ancient martian surface, water-saturated sediment or rock with the radioisotope content of Mawrth Vallis could have supported 1.6 times as many cells in the same volume of water as water-saturated sediment or rock with the radioisotope composition of Melas basin (assuming the same physical properties at Mawrth Vallis and SW Melas). Larger variations in the number of cells may have occurred if the dominant sediment and/or rock at the sites differed in porosity and fracture width, and/or varied in radionuclide concentration on distance scales shorter than the GRS footprints. The physical properties of each location, that is, porosity and fracture geometry, lead to order-of-magnitude variations in H_2_ production. A variety of geological formations likely occur at each of the 11 sites, leading to variations in porosity, fracture width, radionuclide concentrations, and consequently, radiolytic H_2_ production rates.

### 4.2. Total range of radiolytic H_2_ production rates

Calculated H_2_ production rates based on surface martian radioisotope concentrations differ by a factor of ∼6 when ice-covered regions are included (Table 2). The distribution of H_2_ production is principally driven by U and Th series nuclide distributions (Fig. 1) since both decay series produce α-radiation, which carries the most energy and generates the most H_2_ per MeV absorbed. U (^238^U+^235^U) decay series activity and ^232^Th decay series activity, respectively, are responsible for 49–53% and 39–42% of calculated H_2_ production for each site, while ^40^K decay contributes only 4–11%.

The largest production rate, 1.2 n*M*/year for 5% sediment porosity, is associated with Acidalia Planitia in the northern highlands due to this area's high radionuclide abundance (Fig. 2 and Table 2). Acidalia Planitia contains plains of eolian deposits, as well as cone and dome structures possibly formed by mud volcanism, hot spring/geysers, or lava flows (Farrand *et al.*, 2005). If mud volcanism was responsible for the cones, relatively unaltered sediment from depth was transported to the surface. This once-fluid-rich material could contain biomarkers (Farrand *et al.*, 2005; Oehler and Allen, 2010). In general, the northern highlands are enriched in K and Th (Fig. 2). This region contains andesite, basaltic andesite, or weathered basalt (Bandfield, 2000; Wyatt and McSween, 2002). Basaltic lava flows at Tharsis Montes and Olympus Mons shield volcanoes have below average K and Th concentrations.

Just as radionuclide-enriched regions yield the maximum calculated H_2_ production rate, radionuclide-depleted regions yield the minimum rate (Fig. 2). Between 75°N and 75°S, Promethei Terra yields the lowest calculated rate, 0.33 n*M*/year for 5% porosity sediment (Table 2). Promethei Terra consists of interbedded volcanic rock and impact breccia. There is evidence that both fluvial and glacial processes shaped the terrain of Promethei Terra. The landscape of Promethei Terra was also shaped by the impact that formed the Hellas basin (Ivanov *et al.*, 2010). The radioisotope composition of the large impact basin, Hellas Planitia, also yields a low calculated H_2_ production rate: 0.44 n*M*/year for 5% porosity sediment (note there is high uncertainty in GRS measurements in Hellas Planitia, resulting in ∼17% relative uncertainty of the H_2_ production rate).

The calculated production rates for Mars' northernmost latitudes (*e.g.*, 0.21 n*M*/year for 5% porosity sediment) are lower than those associated with Promethei Terra, which has higher radionuclide concentrations (Table 1). As previously mentioned, the measured concentrations are likely biased by the permanent ice cap. Despite the bias toward low values for this region covered by water ice and dust (Bibring *et al.*, 2004), calculated H_2_ production can reach rates higher than those derived for ice-free regions of Mars; the maximum rate calculated for sediment of the ice-capped area is ∼0.6 n*M*/year. This comparison suggests that there may be locally high H_2_ generation rates within or under the polar ice caps. However, H_2_ *G*-values for ice are about half of those for liquid water used in our study (Table 3). *G*(H_2_) values for α-, β-, and γ-radiation in ice at temperatures of 77 K are 0.7, 0.3, and 0.1 molecules H_2_/100 eV, respectively (Johnson and Quickenden, 1997).

### 4.3. Comparison with terrestrial H_2_ production rates

Calculated H_2_ production rates for martian microfractured rock overlap with calculated rates in Earth's basaltic seafloor. In the upper 100 m of South Pacific basalt, production rates range from ∼0.02 to 0.6 n*M*/year (Dzaugis *et al.*, 2016). Calculated martian production rates for water-saturated low-porosity sediment with the radioisotope composition of the Mars 2020 sites generally exceed those of South Pacific basement basalt. Rates for water-saturated, high-porosity martian sediment (≥35%) and water-filled martian microfractures (1 μm wide) are comparable with South Pacific basalt rates. Rates for larger water-filled martian fractures (1 cm wide and 10 cm wide) are generally one to two orders of magnitude lower than South Pacific basalt rates (compare with Dzaugis *et al.*, 2016).

Radiolytic H_2_ has been suggested to be the dominant electron donor in Earth's subseafloor basalt older than 10 Ma (Türke *et al.*, 2015; Dzaugis *et al.*, 2016). On Earth, microbes have been isolated from subseafloor basalt as old as ∼71 Ma (Sylvan *et al.*, 2015).

Although these data from Earth are intriguing in their implications for life on Mars, we do not yet have enough information to definitively calculate how much biomass consumption of radiolytic H_2_ might support on Mars. For example, the Gibbs energy of martian H_2_ consumption is not yet known, because we do not know *in situ* concentrations of H_2_ or relevant oxidants.

### 4.4. Enhanced H_2_ production

The martian rates we calculate are based on bulk GRS measurements for the 11 locations. At finer spatial scales, radiolytic H_2_ production rates may locally be much higher or lower, as the distribution of radionuclides is not homogeneous throughout all sediment and basalt. In Earth's oceanic crust, Türke *et al.* (2015) calculated radiolytic H_2_ production within palagonite, altered basaltic glass enriched in radionuclides and containing ∼14–38 wt % H_2_O (Pauly *et al.*, 2011). This combination of high radionuclides and water content can result in high H_2_ production rates. Türke *et al.* (2015) calculated H_2_ accumulation rates in palagonite basalt samples taken from North Pond Area, ridge flack of the Mid-Atlantic Ridge, using radiolysis models based on Blair *et al.* (2007) and Lin *et al.* (2005a). Their calculated rates are between 0.15 and 1.75 n*M*/year in the rocks' intergranular water. Given fluid flow and estimates of H_2_ concentrations at North Pond, Türke *et al.* (2015) calculate that radiolytic H_2_ can accumulate to levels high enough in the ridge flank to support hydrogentrophic life.

On Mars, there is abundant evidence of the presence of hydrated silicate and sulfate minerals that may provide habitable environments for microorganisms (Ehlmann *et al.*, 2009). Hygroscopic sulfate salts have also been detected in martian soils (Smith *et al.*, 2014). Through deliquescence, these salts can produce saturated brines with very low freezing points (Davila *et al.*, 2010; Al Soudi *et al.*, 2017). If the hygroscopic sulfate salts contain radionuclides, this may produce another habitable microenvironment for microorganisms that obtain energy from sulfate reduction by H_2_. Microscale variation aside, the mean rates in bulk wet martian sediment and microfractures are capable of supporting microbial life.

H_2_ yields may locally be higher than those we have calculated, because some minerals catalyze radiolytic H_2_ production. For example, mordenite, a zeolite mineral, increases *G*(H_2_) values for γ-radiation relative to pure water by up to a factor of ∼3 (Kumagai *et al.*, 2013). There is spectral evidence of zeolite minerals on Mars' surface (Ruff, 2004; Ehlmann *et al.*, 2009).

Bromine-enriched brine also catalyzes radiolytic H_2_ production, relative to pure water; LaVerne *et al.* (2009) experimentally showed that Br-saturated solutions have higher *G*(H_2_) values for γ radiation than pure water. The martian surface contains evidence of past and present brines (Rao *et al.*, 2005; Martínez and Renno, 2013). Known Br concentrations on Mars are generally low (ppm) (Rao *et al.*, 2005); however, if Br-rich brines occur or occurred anywhere on Mars, they locally enhance (or enhanced) radiolytic H_2_ production.

## 5. Conclusion

Calculated radiolytic H_2_ production rates (n*M*/year) vary by a factor of 2 for water-saturated sediment or rock with the radioisotope concentrations of sites currently or previously under consideration as the Mars 2020 landing site. The highest calculated rates are for material with the radioisotope concentrations of Acidalia Planitia (where surface materials are enriched in U and Th).

Calculated rates for wet low-porosity martian sediment consistently equal or exceed rates previously calculated for South Pacific basement basalt. Rates for microfractured rock (with 1 μm-wide fractures) and sediment with >35% porosity at the Mars 2020 sites match the low-end of rates calculated for South Pacific basement basalt. In short, radiolytic H_2_ production rates in wet martian sediment and microfractured rock are comparable with rates in terrestrial regions known to harbor microbial life. Consequently, local variation in radiolytic H_2_ production of the ancient wet martian surface will be fruitful to consider during final selection and exploration of the Mars 2020 landing site.

## Supplementary Material

Supplemental data

## Abbreviations Used

GCR: galactic cosmic rays
GRS: γ ray spectrometer
LET: linear energy transfer

## Notes

Associate Editor: Christopher McKay
