# Supplementary material for: Radiolytic H2 Production in Martian Environments
Source: Astrobiology. 2018 Sep 12;18(9):1137–46. doi: 10.1089/ast.2017.1654 (PMC6150936; doi:10.1089/ast.2017.1654)
Supplement: Supplemental data [file Supp_Data.pdf]

Supplementary Data

Supplementary Table S1. Summary of Variables and Parameters Used for the Fractured Hard-Rock Calculations

Variable	*Description*	*Source*
P_{H2}	Total hydrogen yield from radiolysis (nanoMolar H_2/year).	Equation in this article; more details in Dzaugis *et al.* (2015)
$D_{w,i}$	Radiation dose absorbed by water for each radiation type ($i=\alpha$, β, or γ) [MeV/(s·$cm^3{}_{water}$)]	More details in Dzaugis *et al.* (2015)
$G(H_2)_i$	Radiation chemical yield for each radiation type ($i=\alpha$, β, or γ) (H_2 molecules/MeV)	Essehli *et al.* (2011) (α and γ) Kohan *et al.* (2013) (β)
A	Activity of each nuclide for each decay series, ^{238}U, ^{235}U, ^{232}Th, and ^{40}K [decays/(s·g_{rock})]	Boynton *et al.* (2007) GRS data
ρ_r	Rock density (g/cm^3)	Baratoux *et al.* (2014)
E_0	Initial radiation energy (MeV/decay)	Nuclide Datasheets from Nucleonica GmbH, 2014
R_{stop}	Stopping distance, the maximum distance traveled by charged particles (μm)	NIST database (Berger *et al.*, 2005; Hubbell and Seltzer, 2004) was used for all calculations except α-particles through basalt from Brennan and Lyons (1989)
b	Parameter used in the attenuation equation of charged particles, determined by the slope of energy–range relationships (see Supplementary Table S3)	NIST database (Berger *et al.*, 2005; Hubbell and Seltzer, 2004) was used for all calculations except α-particles through basalt from Brennan and Lyons (1989)
μ	Attenuation coefficient for γ-radiation (cm^{-1})	Hubbell and Seltzer (2004)
w	Distance in water from rock–water interface distance (μm)	Fracture widths used in this study–1 μm, 1 cm, and 10 cm

Supplementary Table S2. Summary of Variables and Parameters Used for the Sediment Calculations

Variable	*Description*	*Source*
$P_{H2,sed}$	Total hydrogen yield from radiolysis normalized to sediment volume [Nanomoles H_2/($cm^3 \cdot$year)]	Blair *et al.* (2007)
P_{H2}	Total hydrogen yield from radiolysis. Calculated from $P_{H2,sed}$ and porosity (nanomolar H_2/year).	Blair *et al.* (2007) Equation A2.1
$D_{w,i}$	Radiation dose absorbed by water for each radiation type [MeV/($s \cdot cm^3{}_{water}$)]	Blair *et al.* (2007) Equation A1.5
$G(H_2)_i$	Radiation chemical yield for each radiation type ($i = \alpha$, β, or γ) (H_2 molecules/MeV)	Essehli *et al.* (2011) (α and γ) Kohan *et al.* (2013) (β)
ρ_r	Grain density (g/$cm^3{}_{rock}$)	Baratoux *et al.* (2014)
A_x	Activity of each nuclide for each decay series, where x is ^{238}U, ^{235}U, ^{232}Th, and ^{40}K [decays/($s \cdot g_{rock}$)]	Boynton *et al.* (2007) GRS data
$\Sigma E_{i,x}$	Sum of energy released by each radionuclide decay series and ^{40}K (MeV/decay series)	See Supplementary Table S4
S_i	Relative stopping power of each radiation type ($S_\alpha = 1.5$, $S_\beta = 1.25$, $S_\gamma = 1.14$)	Aitken (1985)
φ	Porosity (volume water/volume sediment)	Porosity values used in this study—5%, 35%, and 80%

Supplementary Table S3. b-Values Used for the Fractured Hard-Rock Attenuation Equations

Radiation type	*Material*	*Energy (MeV)*	b
α	Basalt	3–9	1.46
	Water	2–9	1.45
		<2	0.81
β	Basalt	0.02–0.45	1.68
		0.45–4.5	1.16
	Water	0.01–0.45	1.70
		0.45–4.50	1.17

Supplementary Table S4. Energy Sums for α-, β-, and γ-Decay

	α-Decay energy sum (MeV/decay)	*β-Decay energy sum (MeV/decay)*	*γ-Decay energy sum (MeV/decay)*
^{238}U-series	56.68	2.307	5.834
^{235}U-series	55.01	1.353	1.427
^{232}Th-series	39.10	1.290	4.276
^{40}K	—	0.3502	0.1606

Calculation of these paramentersis based on values from nuclide data tables (Nucleonica GmbH, 2014).

Supplementary Table S5. Fractured Hard-Rock H_2 Production Rates with Uncertainty

	H_2 production rates with SE for water-filled rock fractures (nMH_2/year)		
Site	*Width: 1 μm*	*Width: 1 cm*	*Width: 10 cm*
Acidalia Planitia	0.35 ± 0.02	8E-03 ± 2E-04	3E-03 ± 1E-04
Mawrth Vallis	0.27 ± 0.02	7E-03 ± 2E-04	3E-03 ± 1E-04
**Columbia Hills**	**0.23 ± 0.01**	**6E-03 ± 2E-04**	**2E-03 ± 1E-04**
**NE Syrtis Major**	**0.22 ± 0.02**	**6E-03 ± 2E-04**	**2E-03 ± 1E-04**
**Jezero Crater**	**0.22 ± 0.02**	**6E-03 ± 2E-04**	**2E-03 ± 1E-04**
Nili Fossae	0.21 ± 0.01	5E-03 ± 2E-04	2E-03 ± 1E-04
Holden Crater	0.20 ± 0.01	5E-03 ± 2E-04	2E-03 ± 1E-04
Eberswalde Crater	0.19 ± 0.01	5E-03 ± 2E-04	2E-03 ± 1E-04
SW Melas Basin	0.17 ± 0.01	5E-03 ± 2E-04	2E-03 ± 1E-04
Promethei Terra	0.10 ± 0.01	3E-03 ± 2E-04	1E-03 ± 1E-04
Northern pole	0.06 ± 0.01	2E-03 ± 1E-04	<0.001

Production rate uncertainties are given as standard error (SE). H_2 production rates at each site are associated with the smoothed gamma ray spectrometer radionuclide data (Boynton *et al.,* 2007). Results are given for three fracture widths. **Bold** font marks the current sites under consideration for Mars 2020 rover landing.

Supplementary Table S6. Sediment H_2 Production Rates with Uncertainty

	H_2 production rates with SE for water-saturated sediment (nMH_2/year)		
Site	*Porosity: 5%*	*Porosity: 35%*	*Porosity: 80%*
Acidalia Planitia	1.2 ± 0.1	0.71 ± 0.03	0.19 ± 0.01
Mawrth Vallis	0.93 ± 0.06	0.56 ± 0.03	0.15 ± 0.01
**Columbia Hills**	**0.80 ± 0.05**	**0.48 ± 0.03**	**0.13 ± 0.01**
**NE Syrtis Major**	**0.75 ± 0.05**	**0.45 ± 0.03**	**0.12 ± 0.01**
**Jezero Crater**	**0.75 ± 0.05**	**0.45 ± 0.03**	**0.12 ± 0.01**
Nili Fossae	0.72 ± 0.05	0.43 ± 0.03	0.11 ± 0.01
Holden Crater	0.67 ± 0.04	0.40 ± 0.03	0.10 ± 0.01
Eberswalde Crater	0.65 ± 0.05	0.39 ± 0.03	0.10 ± 0.01
SW Melas Basin	0.58 ± 0.04	0.35 ± 0.02	0.09 ± 0.01
Promethei Terra	0.33 ± 0.05	0.20 ± 0.03	0.05 ± 0.01
Northern pole	0.21 ± 0.03	0.12 ± 0.02	0.03 ± 0.004

Production rate uncertainties are given as standard error (SE). H_2 production rates at each site are associated with the smoothed gamma ray spectrometer radionuclide data (Boynton *et al.,* 2007). Results are given for three sediment porosities. **Bold** font marks the current sites under consideration for Mars 2020 rover landing.

Supplementary References

Aitken, M.J. (1985) *Thermoluminescence Dating*. Academic Press, Orlando, FL.

Baratoux, D., Samuel, H., Michaut, C., Toplis, M.J., Monnereau, M., Wieczorek, M., Garcia, R., and Kurita, K. (2014) Petrological constraints on the density of the Martian crust. *J Geophys Res Planets* 119:1707–1727.

Berger, M.J., Coursey, J.S., Zucker, M.A., and Chang, J. (2005) *ESTAR, PSTAR, and ASTAR: Computer Programs for Calculating Stopping-Power and Range Tables for Electrons, Protons, and Helium Ions (version 1.2.3)*. National Institute of Standards and Technology, Gaithersburg, MD. [Online] Available online at http://physics.nist.gov/Star [2016, June].

Blair, C.C., D'Hondt, S., Spivack, A.J., and Kingsley, R.H. (2007) Radiolytic hydrogen and microbial respiration in subsurface sediments. *Astrobiology* 7:951–970.

Boynton, W.V., Taylor, G.J., Evans, L.G., Reedy, R.C., Starr, R., Janes, D.M., Kerry, K.E., Drake, D.M., Kim, K.J., Williams, R.M.S., Crombie, M.K., Dohm, J.M., Baker, V., Metzger, A.E., Karunatillake, S., Keller, J.M., Newsom, H.E., Arnold, J.R., Bruckner, J., Englert, P.A.J., Gasnault, O., Sprague, A.L., Mitrofanov, I., Squryes, S.W., Trombka, J.I., d'Uston, L., Wanke, H., and Hamara, D.K. (2007) Concentration of H, Si, Cl, K, Fe, and Th in the low- and mid-latitude regions of Mars. *J Geophys Res E Planets* 112:1–15.

Brennan, B.J. and Lyons, R.G. (1989) Ranges of alpha particles in various media. *Ancient TL* 7:32–37.

Dzaugis, M.E., Spivack, A.J., and D'Hondt, S. (2015) A quantitative model of water radiolysis and chemical production rates near radionuclide-containing solids. *Radiat Phys Chem* 115:127–134.

Essehli, R., Crumière, F., Blain, G., Vandenborre, J., Pottier, F., Grambow, B., Fattahi M., and Mostafavi M. (2011) H_2 production by γ and He ions water radiolysis, effect of presence TiO2 nanoparticles. *Int J Hydrogen Energ* 36: 14342–14348.

Hubbell, J.H. and Seltzer, S.M. (2004) *Tables of X-Ray Mass Attenuation Coefficients and Mass Energy-Absorption Coefficients (version 1.4)*. National Institute of Standards and Technology, Gaithersburg, MD. [Online] Available online at http://physics.nist.gov/xaamdi [2016, June].

Kohan, L.M., Sanguanmith, S., Meesungnoen, J., Causey, P., Stuart, C.R., and Jay-Gerin, J. (2013) Self-radiolysis of tritiated water. 1. A comparison of the effects of 60Co γ-rays and tritium β-particles on water and aqueous solutions at room temperature. *RSC Adv* 3:19282.

Nucleonica GmbH. (2014) *Nuclide Datasheets*. Nucleonica Nuclear Science Portal (www.nucleonica.com), Version 3.0.49, Karlsruhe.
